# How therapists in cognitive behavioral and psychodynamic therapy reflect upon the use of metaphors in therapy: a qualitative study

**DOI:** 10.1186/s12888-022-04083-y

**Published:** 2022-06-27

**Authors:** A Malkomsen, JI Røssberg, T Dammen, T Wilberg, A Løvgren, R Ulberg, J Evensen

**Affiliations:** 1 Division of Mental Health and Addiction, Oslo University Hospital, Nydalen, P.O. box 4959, N-0424 Oslo, Norway; 2 Institute of Clinical Medicine, University of Oslo, Blindern, P.O. box 1171, 0318 Oslo, Norway; 3 Department of Psychiatry, Diakonhjemmet Hospital, Vinderen, Box 85, 0319 Oslo, Norway; 4 Nydalen Outpatient Clinic, Nydalen, P.O. box 4959, N-0424 Oslo, Norway

**Keywords:** Major depressive disorder, Cognitive behavioral therapy, Psychodynamic therapy, Metaphor, Qualitative research, Thematic analysis

## Abstract

**Background:**

Research suggests that metaphors are integral to psychotherapeutic practice. We wanted to explore how 10 therapists reflect upon the use of metaphors in therapy, and how they react to some metaphors expressed by patients treated for of major depressive disorder (MDD).

**Methods:**

Five therapists practicing psychodynamic therapy (PDT) and five practicing cognitive behavioral therapy (CBT) were interviewed with a semi-structured qualitative interview. Transcripts were analyzed using a thematic analysis approach.

**Results:**

Our analysis resulted in two main themes: the therapeutic use of metaphors, and conflicting feelings towards metaphors used by depressed patients. Most therapists said that they do not actively listen for metaphors in therapy and many said that they seldom use metaphors deliberately. While PDT-therapists appeared more attentive to patient-generated metaphors, CBT-therapists seemed more focused on therapist-generated metaphors. Most therapists did not try to alter the patient-generated metaphors they evaluated as unhelpful or harmful. Some therapists expressed strong negative feelings towards some of the metaphors used by patients. PDT-therapists were the most critical towards the metaphor of tools and the metaphor of depression as an opponent. CBT-therapists were the most critical towards the metaphor of surface-and-depth.

**Conclusions:**

These results remind us of the complexity of using metaphors in therapy, and can hopefully be an inspiration for therapists to reflect upon their own use of metaphors. Open therapeutic dialogue on the metaphor of tools, surface-depth and depression as an opponent may be necessary to avoid patient-therapist-conflicts.

**Trial registration:**

Clinical Trial gov. Identifier: NCT03022071. Date of registration: 16/01/2017.

## Background

In the book “Man and his symbols”, C.G. Jung wrote about one of his dreams that “it did not state the situation directly but expressed the point indirectly by means of a metaphor that I could not at first understand” [1]. Trying to understand metaphors and their therapeutic value has been of importance to the field of psychotherapy since its very beginning. Lakoff and Johnson theorize that the meaning we ascribe to abstract concepts is based on the way our thoughts are structured in terms of metaphorical concepts – a theory called Conceptual Metaphor Theory (CMT) [2]. The therapeutic relevance of this is clear: If metaphors are important in structuring our thoughts about ourselves and the world, and act as filters that regulate how we view our present and our past – as claimed by Siegelman in his book “Metaphor and meaning in psychotherapy” [3] – they can be an important target of therapy.

There are at least five reasons why therapists should be skilled in using metaphors. First, metaphors appear to be common in therapy [4], and depressed patients both produce and understand metaphors in the same way as people who are not depressed [5]. Second, neuroradiological research has shown that metaphors engage us emotionally in a way that literal language does not [6, 7]. Third, metaphors can help build a therapeutic relationship [8, 9]. Fourth, metaphors seem to motivate us in a way that literal language does not [10, 11]. Fifth, a change in patient metaphors may represent important therapeutic change [12–14].

Metaphors are common in the CBT-nomenclature, and using metaphors is often a part of the CBT training [15]. In their book on CBT-metaphors, Stott et al. point out that metaphors may act as a bridge between the abstract and the concrete in CBT [15]. The book offers numerous examples of metaphors that can be used for psychoeducation (e.g. “worry-thoughts are like quicksand”). Killick et al. interviewed experienced CBT-practitioners about which metaphors they found helpful in therapy, and most of the chosen metaphors were used by the therapists in a psychoeducational manner [16]. Mathieson et al. have shown that CBT-therapists frequently use metaphors, often for the purpose of psychoeducation – with therapists using metaphors twice as often as their patients in recorded sessions [4].

To the best of our knowledge, the focus of the PDT-literature is mostly on the metaphors used by patients. Our impression is in line with a review of the psychodynamic literature on metaphors done in a thesis by Enckell [17], identifying only two authors who focused on therapist metaphors [18, 19]. A common view in the field of psychodynamic psychotherapy is that metaphors make it possible to express something that is difficult for the patient to convey in literal language [20]. In fact, it has been suggested by Borbely that the main concepts in psychodynamic and psychoanalytic theory (e.g. transference, defense) can be described, and even better understood, in figurative terms – “as figuratively organized grammatic entities of the mind’s language” [21].

The research on how therapists reflect upon the use of metaphors in therapy is scarce. However, a recent study by Řiháček et al. provides insight into the differences between therapists in their metaphorical conceptualization of therapy, and the importance of their theoretical orientation [22]. They explored which metaphors psychotherapists used to conceptualize the therapeutic relationship, and identified three underlying metaphors: The Mentor, the Resource Supplier and the Remedy Distributor. Interestingly, they found that The Mentor was significantly more popular in the group of therapists with a cognitive/behavioral orientation than in the group with a psychodynamic orientation. The authors speculate that the difference can be explained by the “more directive nature” of the cognitive/behavioral orientation and the “relative reluctance of insight-oriented therapists to provide their clients with explicit instructions”. This clearly indicates that the theoretical orientation of therapists can influence their preferred metaphors.

To the best of our knowledge, no previous research has focused on how CBT and PDT therapists reflect upon using metaphors in the therapy room. Neither have we found any studies exploring how therapists react to metaphors used by depressed patients. Given the significance of metaphors in therapy, and the inherent ambiguity of metaphors, we believe it is important to shed light on this topic. Thus, we have explored how ten therapists reflect on the topic of metaphors in therapy. To identify any similarities or differences between CBT and PDT therapists, we included five therapists from each therapeutic approach. Our main research question was: How do therapists reflect upon the use of metaphors in therapy and how do they understand and value some of the patient metaphors we identified in our previous study?

## Methods

### Design, ethics and data collection

The present study took place at two public psychiatric outpatient clinics in Oslo, Norway, treating patients with a wide range of mental illnesses. The clinics are part of the specialist health care system and require that patients are referred by a doctor. All therapists were recruited from these clinics.

The study is part of the ongoing Norwegian project on Mechanism of Change in Psychotherapy (MOP) [23]. The aim of MOP is to examine moderators and mediators in CBT and PDT for patients with MDD to develop a better understanding of what works for whom and how. The patients were randomized to either CBT or PDT. The CBT consisted of 16 weekly sessions followed by three monthly booster sessions, and the PDT consisted of 28 weekly sessions.

The Central Norway Regional Ethics Health Committee (REC South East 2016/340) approved the MOP-study, including the qualitative interviews. Clinical Trial gov. Identifier: NCT03022071. Informed consent was obtained from all participants.

### Participants

Therapists were recruited from the MOP-project and invited to a qualitative in-depth interview. All therapists currently treating patients in the MOP-project were asked to participate, and they all agreed. A total of 10 therapists were included, 9 females and 1 male, all between 40 – 60 years old. The therapists were specialists in psychiatry, specialists in clinical psychology or experienced nurses specialized in mental health work. Five of the therapists were formally trained as CBT-therapists, and five were formally trained as PDT-therapists. They all had at least 2 years of formal education in PDT or CBT, in addition to several years of clinical practice. They also attended a 1-year training program to provide PDT or CBT in this project.

### The interview

A semi-structured interview was designed specifically for exploring how the therapists reflect upon metaphors. The interviews were performed by the first author (four interviews) and two research assistants (three interviews each). There were no personal or professional connections between the therapists and interviewers. A research assistant transcribed the interviews and anonymized all the transcriptions. Before the interviews were conducted, the therapists were told that the interview was going to be on the topic of metaphors. We defined the concept of a metaphor as a figure of speech in which a word or phrase literally denoting one kind of object or idea is used in place of another, like “keeping the wheels turning” can mean to “secure the daily operations of outpatient clinic during the pandemic”.

The therapists were first questioned about metaphors in general, and then asked about some of the metaphors we identified in our previous study [24]. We included the metaphors we found to be most common. Examples of questions are found in Table 1.

**Table 1:** Examples of questions from the interview with therapists

Questions about metaphors in general	Questions about specific metaphors
Do you actively listen to the metaphors patients use?	Some patients say they want to get “tools” in therapy – what do you think this is about? What do you think “tools” represent in therapy?
How do you explore what patients mean when they use metaphors?	Some patients say that they want to go “deep” or “deeper” in therapy. How do you understand this wish to go “deep”?
How do you use metaphors in therapy yourself?	Some patients experience depression as a voice or a monster inside them. What do you think the patients mean when they say this?
Can you give some examples on metaphors that you find useful in therapy?	Some patients say that improvement from depression is like taking a journey from darkness to light. What do you think about that?
Have you experienced that patients use metaphors you think are unhelpful or harmful – and did you try to change or correct these metaphors?	What do you think the patients mean when they talk about “chemistry” between therapist and patient?

### Treatment-protocols

The treatment protocols that therapists used to treat patients in the MOP-project were not made with metaphors specifically in mind. The principles of therapy in the CBT-group were based on the book “Cognitive Therapy of Depression” by Aaron Beck et al. [25]. All CBT-therapists made a case formulation based on cognitive principles together with their patients. The therapists were told to inform patients on how to recognize negative, automatic thoughts; examine evidence for and against these thoughts; substitute unhelpful thoughts; recognize the connections between cognition, affect and behavior; and identify and alter dysfunctional beliefs. Therapists were supposed to be continuously active and facilitate collaboration with the patient. The therapy should be focused on the “here-and-now” and limited attention should be paid on recollecting the past [25].

The principles of therapy in the PDT-group were built on the general psychodynamic principles described in the book “Long-term psychodynamic psychotherapy” by Glen O. Gabbard [26]. The time-limited design was built on the principles described by Høglend et al. [27]. All PDT-therapists were asked to make a case formulation together with their patients based on PDT-principles. Therapists were encouraged to explore sensitive topics, explore the patient-therapist relationship, address transactions in the relationship, use material about interpersonal relationships outside therapy as the basis for interventions, encourage exploration of thoughts and feelings about the therapy, and interpret direct manifestations of transference with moderate intensity [28].

### Analysis

We used the method of thematic content analysis [29] to analyze the material. This is a method suited to analyze the experiences and meaning-making of the participants. We identified themes or patterns within the data by using an inductive bottom up-approach – not trying to fit the data into a pre-existing frame [30]. As therapists´ opinions often conflict, we chose to include diverse and contrasting experiences in the results.

The first, second and last author read all the transcripts looking for answers to the question: How do therapists reflect upon metaphors in therapy and how do they react to the patient metaphors identified in our previous study [24]? The first author familiarized himself with the data. He then generated initial codes and searched for themes. The second and the last author read all the transcripts and gave feedback on the first author’s reduction and thematic categorization. The first author then sent the reduced and categorized material to all authors. All authors discussed their unique understanding of the material, and criticized the first author’s categorization. The first author then reorganized the material after getting feedback from the other authors. Finally, all authors agreed on the current categorization and presentation. This process made our interpretations less dependent on individual preferences [31].

Before we conducted the interviews, we speculated that there would be some differences between the CBT and PDT therapists in how they reflected upon the metaphors, but we did not have any pre-formed opinions on what these differences might be. As metaphors are common and not specific to any therapeutic approach, we were also open to the possibility that there might not be any significant differences between the groups. This view was supported by our previous article, where no obvious differences in the use of metaphors were found between patients who received CBT or PDT [24].

The authors have different therapeutic orientations. J.E., T.D. and J.I.R. are CBT-therapists, T.W. and R.U. are PDT-therapists. None of the therapists have any formal training specifically focused on using metaphors in therapy. A.L. and A.M. have no specific therapeutic orientation or training in how to use metaphors in therapy. This diversity may hopefully have broadened our interpretation of the material. We make this transparent in accordance with the checklist of reporting qualitative research by Tong et al. [32].

We wanted to indicate the recurrence and representativeness of therapists’ experiences by using the labels general, typical and variant as suggested by Hill et al. [33]. When something is mentioned by all or all but one therapist it is labeled as general, in the text referred to as “all therapists”. Something is considered typical when it is mentioned by half or more than half the therapists, in the text referred to as “most therapists”. We use the expression “some therapists” when something is found to be a variant represented by less than half the therapists, either in both groups or one of the groups (if specified). The abbreviations CBT and PDT will be used to specify the therapist’s approach. When no abbreviation is used, it means that all therapists in both groups are included.

## Results

We organized the material into two main themes concerning “the therapeutic use of metaphors” and “conflicting feelings towards metaphors used by depressed patients”. In total, ten subthemes were identified. The six subthemes under the first main theme all concern how therapists reflect upon their use, or lack of use, of metaphors in therapy. The four subthemes under the second main theme all concern the conflicting feelings that were evoked in therapists by metaphors used by their patients. The themes and subthemes, including typical quotes from therapists, are summarized in Table 2.

**Table 2:** The identified themes and subthemes with quotes from the therapists

Themes	Subthemes	Examples of quotes
**The therapeutic use of metaphors**	Self-criticism concerning limited awareness and lack of listening	“I don’t explore metaphors specifically. I usually listen to the patient’s own language, but not regarding their metaphors specifically.” (CBT) “I don’t think I have a very conscious awareness of these things (metaphors).” (PDT)
	An arsenal of metaphors or a personalized approach	“I have some metaphors which suit particular situations. I often repeat them because they describe what the patients are struggling with in a nice way.” (CBT) “I wait, I don’t use many metaphors, it’s not like I throw them forward at the first opportunity.” (PDT)
	Strengthening the therapeutic relationship	“It creates a sense of fellowship, our own coded language … It’s something that exists just within our relationship.” (PDT)
	Finding new perspectives and insight	“It can open up a whole other dimension.” (PDT) “I think metaphors can help the patient get out of a rigid way of thinking”. (CBT)
	Exploring metaphors: literal meaning or emotional subtext	“I try to find out if I understand correctly what he is expressing by using a metaphor.” (CBT) “I think it (a metaphor) paves a way into a lot of feelings.” (PDT)
	Unhelpful metaphors: Substituting or reframing	“Some patients hide behind their metaphors.” (PDT) “I seldom correct them, but I may not use their expressions.” (CBT)
**Conflicting feelings towards metaphors used by depressed patients**	Tools: Reassurance or resistance	“It’s an insurance for the patients when you tell them that ‘we have tools, a lot of different tools’”. (CBT) “I can sometimes feel that it invalidates me as a therapist.” (PDT)
	Surface/depth: Different definitions of depth	“I think it’s more about prejudice, that going deep is something they have to do, without realizing what it actually means.” (CBT) “Depth is turn the gaze inward, towards the reactions and feelings.” (PDT)
	Chemistry: Engagement and curiosity	“It’s easier to get good chemistry with people who are interested in therapy.” (PDT) ““That they work in-between the sessions, and don’t just think we can push a button – ‘click!’ – and then it’s over.” (CBT)
	Opponent: Externalizing through metaphors	“It’s a way to externalize some of your own inner forces. (…) It’s easier to blame the depression.” (PDT) “This monster is a metaphor … a way to externalize the depression that can be very fruitful.” (CBT)

### The therapeutic use of metaphors

#### Self-criticism concerning limited awareness and lack of listening

Most therapists said they do not listen actively for metaphors. Many seemed a bit hesitant to admit this lack of active listening, and their answers were often self-critical. One therapist (PDT) said: “No, I guess I can’t really say that I listen actively for metaphors, but … but, not consciously, really … maybe I will after this (interview).” It seemed that CBT-therapists in general were more focused on the therapist-generated than the patient-generated metaphors. One therapist (CBT) said: “That is difficult for me to answer because I’ve mostly been concerned with my own metaphors (laughs).”

Many therapists seemed surprised to be asked if they used metaphors in therapy. One therapist (PDT) said: “That’s a very interesting question. I have to think about it, because that isn’t something I usually reflect upon”. However, all therapists eventually responded that they use metaphors in therapy. Most said that even though they use metaphors, they seldom use them deliberately. One therapist (CBT) said that she mostly uses metaphors “unconsciously” in therapy. Interestingly, many seemed self-critical about their own use of metaphors. Expressions like “I guess I could have done it more often” (CBT) and “not as often as I would like to” (PDT) were common.

#### An arsenal of metaphors or a personalized approach

Most CBT-therapists had a repertoire of metaphors that they learned during their clinical training. One therapist (CBT) explained: “I use many metaphors concerning worry-thoughts, like ‘do not enter the worry-train’.” Another common metaphor among CBT-therapists was the metaphor of “dark depression glasses”.

All CBT-therapists said they use metaphors to explain something or to exemplify a therapeutic point. One therapist (CBT) said she use metaphors to “illustrate techniques that patients can use”. Another therapist (CBT) said she use metaphors “to exemplify, I think, the phenomena, situation or the conflict”. Two therapists (CBT) used the metaphor of a peg to explain how metaphors could be useful to aid memory. One of these therapists (CBT) explained the importance of metaphors in the following way: “They get a peg to hang things on, and that makes it easier to remember something when they’re in the midst of a situation.” One therapist (CBT) also said she uses metaphors to “get the point across” to the patient more effectively. None of the PDT-therapists mentioned these metaphors, and no metaphors were more common than others in the PDT-group. Using metaphors to explain a therapeutic concept seemed less important to the PDT-therapists.

#### Strengthening the therapeutic relationship

Most PDT-therapists highlighted how metaphors could impact the therapeutic relationship. One therapist (PDT) also emphasized the importance of returning to certain patient-generated metaphors as a way of establishing a common language: “The patient showed me this post on (social media) where a weather-beaten bird is walking outside. Below the picture there is a text saying ´Here I am taking a really stupid walk with my stupid mental illness´. This became something we often came back to. She would just say ´Yep, I’m out taking my stupid walk again´.” This point was also made by a CBT-therapist: “It can create a mutual understanding of a dynamic, a person, a relation or an event – a common language, really.”

For some PDT-therapists, metaphors were also used to explore the dynamics of transference-countertransference in therapy. This was described by one of the therapists (PDT) who treated a man with a military background: “The patient makes me feel like I’m waiting on the country-border in (names a Norwegian municipality). He comes up to me and delivers a formal report. It feels very disconnected. (…) I think that’s a metaphor of his defense – to feel in control and report, not to stay in touch with his feelings or engage in a dialogue. So, I told him about it … It was an image he understood immediately, and that he recognized.”

#### Finding new perspectives and insights

Some therapists said they occasionally use metaphors to get the patient to associate in new ways. One therapist (PDT) said: “There may be a point in using some surprising metaphors. To say something to push them out of their regular thinking-box, and make them stop, think a little and maybe become a bit uncertain or curious.” Another therapist (PDT), referring to the associative and emotional aspects of metaphors, said: “It can open up a whole other dimension. It allows both of us to be more free-flowing.” The same point was also made by some CBT-therapists, but framed in a different way. For example, one therapist (CBT) found metaphors to be an effective way to “help the patient get out of a rigid way of thinking”.

Most therapists in the PDT-group emphasized how patients use metaphors to “express something that is difficult to put into words”. Some therapists (PDT) found that metaphors offer patients a way to express something abstract in a concrete way. One therapist (PDT) said: “For patients it can be a helpful tool to view their situation from the outside, to get another point of view. (…) It can stimulate the imagination.”

#### Exploring patient-generated metaphors: literal meaning or emotional subtext

Many therapists said they explore metaphors by making sure they understand what the patient is really trying to express. Most CBT-therapists focused on clarifying whether they had understood the patient correctly. One therapist (CBT) said: “Sometimes I may reformulate what the patient says, like: ´do you mean, for example …´ Just to make sure I understand him.”

The PDT-therapists seemed to focus mostly on the emotional aspects of the metaphors they explored. One therapist (PDT) said she often used metaphors as a way of exploring the patient´s feelings. She occasionally shared with her patients how their metaphors made her feel, and mentioned one example where a patient referred to his mother as a horse-fly: “I could say to him that ‘Oh, I felt a bit sore when you used that word.’” One therapist (PDT) who treated a patient who called himself “a doormat” because he was often exploited, considered the metaphor to be an introduction to something else: “I wondered what this doormat-metaphor really symbolized for him. (…) We talked about it, and this became a way into his relation to those who abused him in his childhood. (…) When you get into these things and find the actual words, I think you should stay within those actual words. And they usually do. The metaphor disappears, in a way.”

#### Unhelpful metaphors: substituting or reframing

Many therapists in the CBT-group said they rarely correct patient-generated metaphors. One therapist (CBT) said she sometimes substituted the patient’s metaphor with her own metaphor: “I seldom correct them, but I may not use their expressions. Instead, I may launch my own expressions.”

Many of the PDT-therapists said they had tried to correct or change a patient-generated metaphor. One therapist (PDT) said she sometimes try to change metaphors that have become a defense against change: “I think that metaphors can be harmful if they become like a pillow to rest on … ‘I’m like a burnt child or wounded animal.’ … That’s a very good metaphor, and it explains something, but it can also become an inappropriate defense against changing and moving on.” One therapist (PDT) said she never corrects a metaphor directly, but instead tries to question it: “If the patient uses a metaphor to explain something, I may question the metaphor. I use the same metaphor, but I may try to take a different angle.”

Another therapist (PDT) said he often views seemingly unhelpful metaphors as a therapeutic possibility: “Let’s say that an anorectic patient says ‘I have to starve that monster inside me’ – that’s a gift, really! Then we can explore this together … how appropriate is it to think this way, and what alternatives are there?” One therapist (PDT) said she sometimes tries to change the metaphor from within, and explained how she had worked with the metaphor of being “stuck” in the depression: “I may ask if they can try to take a tiny step to the left, or maybe move their toe a little bit to the right, to see if that changes anything.”

### Conflicting feelings towards metaphors used by depressed patients

#### Tools: reassurance or resistance

There was a difference between the PDT and CBT-therapists concerning their views on the metaphor of tools. Most of the CBT-therapists said they found this metaphor to be helpful. They supposed that patients wanted something “tangible” and “useful” when they asked for tools. However, not all the CBT-therapists were comfortable with the metaphor of tools. One of the therapists (CBT) said that the demand for tools made her feel anxious: “When the patients come to me and demand tools, I get anxious. I think that ‘I don’t have any tools!’”. However, the same therapist said that she could meet the demand for tools by reformulating: “I guess I could reformulate the ABC-schema or the cognitive diamond as a kind of tool, like ‘here is a tool to help you sort out your thoughts and feelings’.” Most CBT-therapists thought of their therapeutic techniques as tools.

In contrast to the enthusiasm regarding the metaphor of tools in the CBT-group, most of the PDT-therapists responded with a negative attitude towards the metaphor. Most of PDT-therapists regarded the metaphor of tools as an expression of the patients’ longing for a “quick fix” to their problems. One therapist (PDT) said: “They want a crutch, I guess. That’s another metaphor, like a transitional object. A recipe, a certain way to handle something, like an answer sheet. An easy solution.” The same therapist said the patients may be disappointed by the PDT-therapist: “They expect something that makes them well, that makes them walk out of the tunnel and just wake up and everything is fine. This illusion is one of the first things we have to work with.”

The metaphor also seemed to evoke negative feelings in some of the PDT-therapists. One therapist (PDT) said: “It feels like… yeah (breathes out heavily), I get the wind knocked out of me. I don’t get demotivated, it makes me curious, but I just feel like … it really doesn’t interest me, that kind of approach. (…) I can sometimes feel that it invalidates me as a therapist. (…) Do you really think that’s all I’m here for – to give you some tools? I’m not a carpenter.” Another therapist (PDT) said that patients often ask for tools, and that he interprets this as a form of resistance: “I often tell them to find another therapist (laughing). I'm not saying that, but maybe that’s what I’m thinking. (…) In my experience, when patients say that, it is often an expression of resistance.”

#### Surface/depth: different definitions of depth

Some of the CBT-therapists were skeptical to the notion of depth in therapy. One therapist (CBT) said: “I get a kind of itch. I can get a bit irritated because I think many say that PDT is a much deeper therapy than CBT. Like that somehow implies that it’s better. (…) It’s a misconception that CBT isn’t very deep.” The same therapist explained a “deep” therapy in the following way: “What makes a therapy forceful or deep – or whatever you want to call it – is that it makes you change in a way that you can live with, and that it gives you a better life.” Another therapist (CBT), who also was “irritated” by the metaphor of depth, said that she found CBT to be just as deep as PDT: “It’s a metaphor that PDT has claimed ownership to in an unfair way. (…) It irritates me that CBT is seen as a therapy that doesn’t dig very deep. (…) We don’t stay in the past for a long time, but we do get in contact with important experiences in childhood. When they get in touch with some of their core beliefs about themselves, their rules of living, their negative automatic thoughts and all that … then I think we are deep down, in the self, and how they really think about themselves.”

The metaphor of depth did not seem to evoke any negative associations in the PDT-therapists. One therapist (PDT) explained depth in the following way: “It’s about going deeper into oneself, to understand oneself and the challenges one has … and if we’re lucky: to dive deeper into the relational difficulties.” Yet another therapist (PDT) included a third element in the concept of depth: “To go deeper for me is to work with what occurs … parallel-processes and what occurs in the counter-transference. (…) To recreate something that has been difficult in the past, like a relation, and work with it.”

There were discrepancies between how the therapists themselves conceptualized therapeutic depth and how they imagined that their patients conceptualized it. When asked what they thought “going deep” in therapy meant for the patients, most therapists imagined that they wanted to talk about their childhood or their past experiences. One therapist (CBT) said: “I often get the impression that they want to understand things like ‘what was it in my childhood or my life that made things the way they are’. Sometimes that may be important, but at the same time I think it doesn’t help to realize that ‘my mother or father was too distanced’ or whatever it was. That doesn’t help you handle the depression any different here and now.”

#### Chemistry: engagement and curiosity

When asked what they thought patients meant when they used the metaphor “chemistry”, most therapists said either “to be understood”, “to be liked”, “to feel safe” or “to have trust”. However, when asked what chemistry meant to them as therapists, there were some differences. Many CBT-therapists emphasized the importance of patient engagement in therapy, including in-between sessions. One therapist (CBT) explained the basis for therapeutic chemistry in the following way: “That they work in-between the sessions, and don’t just think we can push a button – ‘click!’ – and then it’s over.”

The PDT-therapists seemed less concerned with what the patients did in-between-sessions. However, like the CBT-therapists, most PDT-therapists seemed to think that patient engagement in therapy was an important part of therapeutic chemistry. One therapist (PDT) said: “It’s easier to get good chemistry with people who are interested in therapy, who put a lot of effort into it and take some responsibility – co-responsibility – and who are actually willing to change.” Some of the PDT-therapists seemed to regard bad chemistry as an interesting therapeutic challenge. For example, one PDT-therapist believed that supposed bad chemistry could actually be good chemistry: “Good chemistry may be something else for me than it is for the patient. I think that there may actually be good chemistry even though the patient feels that the chemistry is bad. (…) If there is space for the patient to actually talk and say things like that … that’s a good climate.”

#### Opponent: externalizing through metaphors

The therapists did not agree on the usefulness of conceptualizing the depression as an opponent, demonstrated by metaphors like depression as a monster or an internal voice. The concept of externalization seemed to be important for both groups of therapists, but their attitude towards it were quite opposite.

Some of the PDT-therapists seemed to dislike the metaphor of depression as a monster and internal voice because they found it to be a way of externalizing the depressive reaction. One therapist (PDT) said the following about depression as an internal voice: “It’s a way to externalize some of your own inner forces. (…) It’s easier to blame the depression.” One therapist (PDT) also found these metaphors to be a defense mechanism, but viewed this as a useful vantage point for exploration: “I think it may be useful – absolutely. (…) We can use this to look at the depression as a defense. That’s really interesting.” Another therapist (PDT) said that the metaphor of an internal voice gave her “some associations to the super-ego”.

Many therapists in both groups seemed to agree that externalization may be necessary in the first phases of therapy. One therapist (CBT) made the following comment on the metaphor of an internal voice: “I think it can be okay as a part of an understanding. But the main goal of all this is to acknowledge that ‘all this is a part of me’.” However, many CBT-therapists also regarded the externalization to be a crucial benefit of metaphors – even in the later stages of therapy. One therapist (CBT) said she “loved” the metaphor of the depression talking: “That’s exactly what I’m trying to get them to do – to externalize a bit.”

## Discussion

This study aimed to explore how therapists in CBT and PDT reflect upon the use of metaphors in therapy and how they react to some of the patient-generated metaphors we identified in our previous study [24]. There are some differences between CBT and PDT-therapists in how they reflect upon the use of metaphors, and these differences seem in large to be in line with the theoretical rationale behind the therapeutic approaches.

Most therapists said they do not actively listen for metaphors in therapy, and many said that they seldom use metaphors deliberately. While PDT-therapists appeared more focused on patient-generated metaphors, CBT-therapists seemed more focused on therapist-generated metaphors. PDT-therapists were the most critical towards the metaphor of tools and the metaphor of depression as an opponent. CBT-therapists were the most critical towards the metaphor of surface-and-depth. These results will now be discussed in more detail.

### A self-critical attitude

Therapists in both groups reported limited awareness of metaphors and said that they seldom actively listen for metaphors. One reason for this could be that the therapists do not believe that metaphors are important or useful, but this does not seem to be the case. In contrast, the therapists said they believed that metaphors are important and seemed self-critical – even a bit hesitant – when they admitted their own lack of active listening and limited awareness.

For the CBT-therapists, this may be a consequence of the fact that much of the literature seem focused on therapist-generated metaphors. For the PDT-therapists, much is written on how they should handle patient metaphors, but there is no consensus. Considering the lack of solid empirical evidence, it is not surprising that therapists seldom agree on best practice. Some think it is important to share the metaphors with patients to create a mutual language [34], while others find it more effective to interpret them [35]. Some have emphasized the importance of recognizing what metaphors reveal about the patient’s defenses and fantasies [36], while others argue that metaphors are therapeutically created to undo symptoms and reveal the creative and integrative operations of the ego [37]. This lack of consensus may create a confusion that may partly explain why many therapists say they do not listen actively for metaphors.

### Therapists’ use of metaphors in CBT

The CBT-treatment manual of our study by Beck et al. does not go into depth on how therapists should relate to metaphors in therapy [25]. Regarding the therapeutic use of metaphors, it simply suggests that the therapist can “increase rapport by reflecting the patient’s feelings back to him in the form of a sensitive summary, analogy, or metaphor”(p.53).

CBT-therapists said they made use of metaphors in ways that could fit with the treating principles of their therapeutic approach. For example, psychoeducation is an important part of the treatment in CBT and metaphors are common in the examples of ways to educate patients in the CBT-literature. The point is often to “build cognitive bridges” by using metaphors [15]. The “Oxford Guide to Metaphors in CBT” offers numerous examples of such cognitive bridges, like “intolerance of uncertainty may be thought of as an allergy” and the stress-vulnerability model explained as “having a bucket with a hole in it” [15].

When the CBT-therapists say they mostly use metaphors to explain a therapeutic concept, this may be a consequence of the treatment principle in CBT about teaching patients new skills (e.g. monitoring negative thoughts or alter dysfunctional beliefs). We cannot conclude from what therapists claim they do in therapy to what they actually do, but our results are in line with the study by Mathieson et al. on metaphor use in CBT [38]. They found that “bursts” of metaphor use initiated by therapists were more likely to be a recurrence of a metaphor previously used by the therapist than of a metaphor previously used by the client [38]. In other words, CBT-therapists seem more focused on their own metaphors than on patient metaphors. It may be that this emphasis on therapist-generated metaphors somewhat hinders the therapists’ responsiveness towards patient-generated metaphors in CBT, but this hypothesis is yet to be empirically studied.

The literature in CBT often highlights the importance of creating a mutual language by using metaphors (e.g. in case formulations) [39]. With the strong emphasis on cooperation between therapist and patient in CBT, which is also mentioned in the treatment manual, it is somewhat surprising that therapists say they seldom co-create metaphors with their patients. The CBT-therapists mention the benefits of a mutual language in strengthening the therapeutic relationship, but seldom mention the possibility of creating a metaphor together with the patient. Studies have shown that training CBT-therapists to co-create metaphors with patients and respond to patient metaphors can enhance the therapeutic alliance [8, 9]. Studies have also shown that a good therapeutic alliance is associated with good outcome [40]. Given that co-creation and attention to patient-generated metaphors seem both beneficial and in line with the theory of CBT, this may be an area of possible improvement for the CBT-therapists.

### Therapists’ use of metaphors in PDT

The book by Gabbard that is used by the therapists to guide the treatment, does not detail how therapists should relate to metaphors in therapy [26]. However, it does state that “capacity to think in terms of analogy and metaphor” is one of the characteristics that are “predictive of a good capacity to use exploratory therapy”(p.32). Metaphor is also mentioned in the context of how therapists should relate to resistance in therapy. The manual states that resistance “connotes an obstacle that must be removed and thus may evoke military metaphors” and that this may make the therapist tempted to launch a “frontal assault” on the resistance (p.99). Instead, Gabbard advises therapists to “regard the resistance as an informative and illuminating revelation about who the patient is”(p.100).

The PDT-therapists in our study say that they seldom use their own metaphors to explain concepts relevant to the therapeutic situation, but instead focus more on the patient-generated metaphors. As outlined in the introduction, much of the literature in PDT is focused on patient-generated metaphors, which may partly explain why PDT-therapists have this as their main focus. When some of the PDT-therapists described patient-generated metaphors as an unconscious defense, this view is reasonable in light of the PDT-literature. For example, a study by Stuart showed that the use of novel figurative language (e.g. metaphors) sometimes accompanied an increase in ratings on the “Patient Experiencing Scale” (EXP), but most often coincided with a decrease [41]. The idea that all defense is metaphorical in its nature is explored extensively by Borbely, who argues that “whether normal or neurotic, defenses are metaphorically or metonymically structured” and uses an example of “overcompensatory love” and “repressed aggression” in which “love comes to stand for hate” [42]. The close relation between metaphor and defense is also mentioned by most of the PDT-therapists we interviewed. The suspicion that patients sometimes hide behind their metaphors to escape difficult emotions, was more prevalent in the group of PDT-therapists than in the CBT-group. This PDT-concept of “defensive metaphors” might also be the reason why some PDT-therapists say they try to alter the patient-generated metaphors they find unhelpful or harmful.

Whether it would be beneficial for PDT-therapists to focus more on therapist-generated metaphors – e.g. in the use of case formulations or psychoeducation – is an empirical question that remains to be answered. We cannot claim that this would be effective in PDT simply because it appears effective in CBT. However, it seems unlikely that PDT-therapists could not learn anything from the literature focused on therapist-generated metaphors in CBT, provided that it is integrated without violating the treating principles of PDT. This may possibly be an area of improvement for PDT-therapists.

### Conflicting feelings towards metaphors used by depressed patients

Patient metaphors like tools, chemistry, depth and depression as an opponent evoke conflicting emotions in the therapists. PDT-therapists expressed negative emotions when they were asked about the metaphor of “tools”. It may be that these negative emotions are triggered because the metaphorical demands by the patients do not match the therapists’ metaphorical ideas about “good” therapy. In general, the PDT-therapists seemed to think that the concept of tools was counterproductive in therapy and that they did not possess the “tools” that patients wanted. However, we could not find any signs that dissatisfaction with the lack of tools was more prominent in patients who got PDT than CBT in our previous study [24]. The “lack of tools” was a critique presented by patients in both groups. Most PDT-therapists said that they may be able to redefine their techniques to fit the metaphorical concept of tools, even though they seemed a bit reluctant to do so. The reason for this hesitation seems to be that they find tools to represent a longing for a “quick fix” – something they do not believe in. Importantly, this presupposition is not supported by our interviews with patients. The patients actually seem to be more flexible than the therapists in their definition of tools. One patient (PDT) said that she got many “tools” in therapy, for example: “accepting myself, and asking the why-question, as she (the therapist) did, but now asking it myself” [24]. This definition of tools seems to fit the treatment manual of PDT quite well. Another patient (CBT) said she used the tools she got in therapy “to build herself up” – a metaphor that does not fit the therapists’ presupposition of patients “longing for a quick fix” [24]. Hopefully, this serves as a reminder not to project our own interpretations of metaphors onto others – but explore.

The therapists’ reactions to the metaphor of depression as an opponent revealed differences between PDT and CBT-therapists in their views on externalizing. In general, the PDT-therapists seemed more skeptical than CBT-therapists to metaphors that made patients separate their depressive feelings from themselves. Thus, the PDT-therapists said that they often avoided these metaphors. CBT-therapists also viewed these metaphors as ways of externalizing, but highlighted this as one of the positive effects of the metaphors. This is in line with the principles of both CBT and PDT. In PDT, externalizing is often seen as an unconscious defense that is “a good indication of lack of awareness”(p.345) [43]. In the CBT, conversely, externalizing is often seen as a healthy way of coping with psychological disorder because it offers the patient to view the disorder “as separate from herself”(p.196) [15].

Interestingly, in our previous study, we did not find any major differences between PDT and CBT-patients in their use of metaphors conceptualizing depression as a disease or opponent [24]. Both PDT and CBT-patients said that therapy had taught them that “this is a disease, in a way, so it’s not my fault” (PDT) and described improvement like “the monster living inside me didn’t have as much authority anymore” (CBT). Overall, our study indicates that the differences in therapists’ reflections upon metaphors do not affect how patients use metaphors to make sense of their therapy – at least not in ways that we were able to uncover by using metaphorical analysis in our previous article and thematic analysis in this article. It may be that other research methods, a larger sample or the same study in another population would come to different results.

### Strengths and limitations

Our study has several limitations. First, we have not used explicit criteria for the definition of conceptual metaphors. This is seldom done in research on conceptual metaphors, and this lack of operationalization has been raised as a major obstacle toward accepting CMT as a comprehensive theory of metaphors [44]. As a result, it is still unclear whether researchers have used similar criteria in their identification of metaphors. Thus, to define conceptual metaphors in more detail is warranted, but beyond the scope of this study. Second, no audio or video from therapy sessions have been evaluated to check whether what the therapists say they do in therapy matches what they actually do. This limits our study to provide knowledge on what therapists claim about their therapeutic practice. Third, we cannot say anything statistically significant about the differences between CBT and PDT; a much larger sample than 10 is normally required for a statistically significant result [45]. Fourth, we do not know anything about the usefulness of metaphors in therapy from the perspective of the patients. This would be important knowledge as many therapists seemed self-critical about their limited use and awareness of metaphors. The usefulness of metaphors from the perspective of the patients is an important focus for future research.

Despite these limitations, we still believe our study provides valuable knowledge on the possibilities and pitfalls of using metaphors in therapy. A major strength of qualitative research is that issues can be examined in detail – providing sufficient room for the many subtleties and complexities of the research subject that is often missed in more positivistic explorations [46]. In the exploration of how therapists reflect upon the use of metaphors in therapy, many complex issues arise. In our opinion, this makes a thematic analysis of ten therapists a fitting design for our study.

However, a small-scale qualitative study examining ten therapists from only two different outpatient clinics, cannot be generalized to all therapists. In addition, a topic such as metaphors will always be sensitive to differences in language and culture [47]. However, our results can still be transferred to other clinicians and researchers, and thus provide important perspectives. The concept of transferability is about the ability of readers to decide how best to apply the results in their own context [48]. Given that most of the differences between the groups are deeply rooted in the literature and traditions of PDT and CBT, we believe that our results will be relevant in other contexts as well.

## Conclusions

Our study is a reminder of the complexity and possible pitfalls of metaphors in therapy, and hopefully an inspiration for therapists to reflect upon their own use of metaphors. Further, an open dialogue with patients concerning the metaphor of tools, surface-depth and depression as an opponent may be necessary to avoid patient-therapist-conflicts.

## Data Availability

The datasets generated during and analyzed during the current study are not publicly available due to the sensitivity of the material and patient safety, but are available from the corresponding author on reasonable request.

## Abbreviations

BDI: Becks Depression Inventory
CBT: Cognitive Behavioral therapy
DSM-V: Diagnostic and Statistical Manual of Mental Disorders—version IV
M.I.N.I.6.0.0: Mini International Neuropsychiatric Interview
MDD: Major Depressive Disorder
MOP: Mechanisms Of change in Psychotherapy
STPP: Short Term Psychodynamic/psychoanalytic psychotherapy
CMT: Conceptual metaphor theory
CCC-RS: The Collaborative Case Conceptualization Rating Scale
WAI-SR: Working Alliance Inventory
